# Microstructure and Mechanical Properties of In Situ Al_3_Zr/Al-5Cu-0.6Mn-0.15Ti Heat-Resistant Aluminum Matrix Composites Based on Nominal Al_3_Zr Contents

**DOI:** 10.3390/ma19132838

**Published:** 2026-07-03

**Authors:** Kaiyan Zhang, Tingting Zhang, Yu Xiong, Chunting Zhang, Jinjin Li, Liwen Pan

**Affiliations:** 1 School of Resources, Environment and Materials, Guangxi University, Nanning 530004, China; 2415301089@st.gxu.edu.cn (K.Z.); nancym1029@163.com (T.Z.); xiongyxy@163.com (Y.X.); 15777058583@163.com (C.Z.); 13977161308@139.com (J.L.); 2 Guangxi Key Laboratory of Processing for Non-Ferrous Metal and Featured Materials, Nanning 530004, China; 3 MOE Key Laboratory of New Processing Technology for Non-Ferrous Metals and Materials, Nanning 530004, China; 4 Key Laboratory of High Performance Structural Materials and Thermo-Surface Processing, Education Department of Guangxi Zhuang Autonomous Region, Guangxi University, Nanning 530004, China

**Keywords:** Al-Cu alloy, in situ reaction, Al_3_Zr reinforced particle, microstructure, elevated-temperature mechanical properties

## Abstract

**Highlights:**

- In situ synthesized D0_23_-Al_3_Zr particles refine α-Al grains and improve the material’s thermal stability.
- T6-treated 4.5 wt.% Al_3_Zr composite achieves optimal tensile strength at RT and 350 °C, increasing by 8.56% and 23.31% vs. the base alloy.
- Al_3_Zr particles retain their morphology and size after 24 h of thermal exposure at 350 °C; their stable dispersion strengthening, together with the smaller degree of θ′-Al_2_Cu coarsening observed, reduces the strength/hardness degradation relative to the base alloy.
- Combined and largely independent contributions from Al_3_Zr particle strengthening and θ′-Al_2_Cu precipitation strengthening account for the overall strengthening and thermal stability of the composite.

**Abstract:**

*x*Al_3_Zr/Al-5Cu-0.6Mn-0.15Ti composites were fabricated via an in situ reaction method, and the influence of Al_3_Zr content on the microstructure and mechanical properties in both as-cast and T6-treated conditions was systematically investigated. The results reveal that the D0_23_-Al_3_Zr content increases in proportion to the K_2_ZrF_6_ addition level. Following T6 heat treatment, finely dispersed θ′-Al_2_Cu precipitates were formed within the matrix, and the α-Al + θ-Al_2_Cu eutectic network dissolved. The blocky Al_3_Zr particles underwent spheroidization and could continuously exert a grain boundary pinning effect to suppress grain coarsening. After T6 heat treatment, the 4.5 wt.% Al_3_Zr composite exhibited average ultimate tensile strengths of 324.44 MPa at room temperature and 123.38 MPa at 350 °C, corresponding to improvements of 8.56% and 23.31%, respectively, relative to the unreinforced base alloy. Following thermal exposure at 350 °C for 24 h, the composite exhibited less pronounced coarsening of the θ′-Al_2_Cu precipitates compared with the base alloy, while the Al_3_Zr particles retained their morphological and dimensional stability. Consequently, the reductions in both tensile strength and hardness were smaller than those observed for the base alloy. Analysis indicates that Al_3_Zr particles significantly refine the α-Al grains and enhance the alloy’s thermal stability. The superior property retention is attributed primarily to the high thermal stability of the Al_3_Zr particles, which preserve their dispersion-strengthening contribution at 350 °C, with the reduced θ′ coarsening as a contributing factor. The overall strengthening of the composite arises from the combined and largely independent contributions of Al_3_Zr particle strengthening and θ′-Al_2_Cu precipitation strengthening.

## 1. Introduction

Aluminum alloys have a density approximately one-third that of conventional steels while exhibiting strength comparable to that of low-carbon steel, making them high-performance, lightweight, and environmentally friendly engineering materials widely used in automotive manufacturing, aerospace, and related industries [1,2]. Among these, Al–Cu alloys exhibit relatively high room-temperature strength, good formability, and significant heat-treatment strengthening potential. Such features make them attractive options for high-performance structural materials. However, above 225 °C, the metastable θ′-Al_2_Cu precipitates rapidly destabilize and coarsen, thereby diminishing the strengthening effect and resulting in rapid softening and failure [3], which severely restricts the use of such alloys in elevated-temperature environments. Consequently, considerable research has been devoted to improving the microstructural stability and elevated-temperature mechanical properties of Al–Cu alloys above 300 °C.

Aluminum matrix composites have gained growing popularity as an effective means of improving the elevated-temperature performance of aluminum alloys. The basic approach involves embedding high-strength, thermally stable particulate reinforcements into the aluminum matrix. Depending on how the reinforcement is introduced, particulate-reinforced aluminum matrix composites fall into two main categories: ex situ addition composites and in situ synthesized composites. The ex situ process involves the direct incorporation of pre-fabricated reinforcement particles, such as SiC [4], Al_2_O_3_ [5], TiC [6], and B_4_C [7], into the aluminum melt through mechanical stirring, powder metallurgy, or squeeze casting. However, the inherently poor wetting of ceramic particles in molten aluminum promotes particle agglomeration and segregation. As a result, achieving a homogeneous dispersion of the reinforcement becomes extremely challenging. At the same time, chemical incompatibility at the reinforcement–matrix interface often leads to weak interfacial bonding, preventing the particles from being fully integrated into the matrix. Because of these combined wetting and bonding problems, the casting route is substantially limited in its ability to produce aluminum matrix composites with high particle volume fractions. In contrast, the in situ reaction approach works by introducing additives into the aluminum melt so that, under elevated temperatures, chemical reactions occur, producing reinforcements such as TiC [8], Al_3_Ti [9], Al_2_O_3_ [10], and Al_3_Zr [11,12] directly within the matrix. In situ-synthesized reinforcements tend to be small, distributed relatively uniformly, and bond strongly to the matrix. These traits make it easier to increase the reinforcement volume fraction while maintaining high composite quality. Because of this, the in situ reaction route is a more attractive option for producing high-performance, high-volume-fraction particle-reinforced AMCs.

The Al_3_M-type strengthening phase formed in situ possesses a lattice parameter very close to that of the aluminum matrix, leading to a minimal lattice mismatch [13]. It establishes a coherent or semicoherent interface with the matrix, which effectively pins dislocations and hinders grain-boundary sliding, thereby enhancing the elevated-temperature strength and stability of the aluminum alloy [14,15,16]. Moreover, binary phase diagrams of the Al-Ti, Al-V, and Al-Zr systems show that Al_3_M-type phases melt at higher temperatures than the aluminum matrix, thereby conferring on them superior thermal stability. Currently, in situ synthesis via solid–liquid reactions enables the formation of equiaxed D0_22_/D0_23_-type Al_3_M phases [17,18,19], which exhibit thermal stability exceeding 500 °C and can reach up to 75% of pure aluminum’s melting point [14,15,16,20]. A considerable body of work has been devoted to adding reactants to aluminum matrices to trigger in situ reactions that generate stable or metastable Al_3_M strengthening phases, thereby improving the elevated-temperature behavior of aluminum alloys. Pandee et al. [11] fabricated A356/Al_3_Zr composites via an in situ reaction between A356 alloy and K_2_ZrF_6_. The study showed that the in situ formed Al_3_Zr reinforcing phases exhibited a block-like morphology and were evenly dispersed within the matrix, thereby refining the α-Al grains and improving the eutectic Si structure. Consequently, a notable improvement in mechanical properties was observed, with the T6-treated sample exhibiting an ultimate tensile strength of 319 MPa. Liu et al. [12] produced Al_3_Zr/Al–Zn–Mg–Cu composites by means of an in situ reaction method. Their work showed that Al_3_Zr particles were effective at pinning grain boundaries, which in turn kept coarse columnar grains from excessive growth. This also lowered the risk of hot cracking. The overall outcome was a shift toward equiaxed grain formation, along with a noticeable improvement in the material’s mechanical properties. Ma et al. [21] produced TiB_2_/Al2618 composites through an in situ reaction route. Their findings indicated that TiB_2_ particles promoted dynamic recrystallization while simultaneously constraining grain growth. The net result was a markedly refined microstructure. Beyond that, these particles also contributed to increased precipitation, thereby improving the mechanical properties of the composites.

Among the different types of in situ generated particles, Al_3_Zr has attracted considerable attention as a promising strengthening phase because of its excellent physical and mechanical characteristics [22], such as low density (4.11 g/cm^3^), high melting point (1580 °C), and superior elastic modulus and thermal stability [23]. Al_3_Zr and the aluminum matrix have only a small lattice mismatch, which helps the two phases bond firmly at the interface. The thermal expansion coefficient of Al_3_Zr (16.0 × 10^−6^ K^−1^) is fairly close to that of the aluminum matrix (23.6 × 10^−6^ K^−1^), so thermal stresses are largely relieved. What is more, Al_3_Zr has extremely low solid solubility in the aluminum matrix, thereby resisting coarsening and dissolution even at high temperatures. Therefore, Al_3_Zr is considered a highly promising reinforcing phase for improving the elevated-temperature properties of aluminum alloys. However, previous studies have primarily focused on the effects of Al_3_Zr in Al–Si alloy systems [24,25]; the effects of incorporating D0_23_-Al_3_Zr into Al-Cu alloy composites remain insufficiently investigated.

In this study, an Al–Cu–Mn–Ti alloy was selected as the matrix material, and K_2_ZrF_6_ salt was introduced into the melt to induce an in situ reaction to form the Al_3_Zr phase. Composites with different Al_3_Zr contents were successfully fabricated, and their microstructures, along with their tensile strengthening behavior at both room and high temperatures, were systematically studied. This research is expected to offer useful theoretical and experimental guidance for the development of new heat-resistant aluminum alloys.

## 2. Materials and Methods

### 2.1. Experimental Materials and Equipment

The raw materials employed in this study included commercial-purity aluminum granules (99.7 wt.%), potassium hexafluorozirconate (K_2_ZrF_6_), Cu (99.9 wt.%), Al-10Mn, and Al-10Ti master alloys. A 50 wt.% KCl–50 wt.% NaCl mixed salt served as the melt covering flux, hexachloroethane (C_2_Cl_6_) as the refining agent, and a coating with a Na_2_SiO_3_:ZnO mass ratio of 1:3 was applied to the crucible.

### 2.2. Alloy Smelting and Preparation

Melting of the alloy was carried out in a graphite crucible placed inside a medium-frequency induction furnace (M.M.F.00008, Yihui Castino, Guangzhou, China) under ambient air. Before melting, the inner surface of the crucible was evenly covered with a refractory coating and allowed to dry completely. Dry aluminum blocks were put in the crucible. An induction power supply heated the crucible until the aluminum blocks were completely melted. A K-type thermocouple tip was inserted into the molten aluminum to measure its temperature, and the heating power was adjusted to maintain a melt temperature of 760–780 °C. Pure copper was added, followed by sequential additions of Al-10Mn and Al-10Ti master alloys, and the temperature was subsequently reduced to 740–750 °C. The covering agent (50% KCl + 50% NaCl mixed powder) was sprinkled onto the melt surface, and the melt was maintained at this temperature for approximately 5 min. After the master alloy had completely melted, the surface covering flux was skimmed off. According to the stoichiometric ratio of reaction equation (1), additional aluminum granules were added to compensate for the aluminum consumed during the in situ reaction. A graphite stirring rod was inserted into the melt, and mechanical stirring was conducted at 600 r/min. Once a vortex formed in the melt, K_2_ZrF_6_ was introduced at 2.47 wt.%, 4.94 wt.%, 7.41 wt.%, and 9.88 wt.% of the total alloy mass (corresponding to nominal mass fractions of Al_3_Zr of 1.5 wt.%, 3 wt.%, 4.5 wt.%, and 6 wt.%, respectively). After stirring for 10 min, an in situ reaction occurred in the melt [11]:

3K_2_ZrF_6_ + 13Al→3Al_3_Zr + 3KAlF_4_ + K_3_AlF_6_ (1)

After allowing the melt to settle, the reaction residues (K_3_AlF_6_ and KAlF_4_) on the melt surface were removed. Degassing was performed twice by introducing C_2_Cl_6_ into the melt at 720 ± 10 °C, with a total addition of 0.5 wt.% of the melt mass, divided equally between two batches. During each degassing step, C_2_Cl_6_ was pressed to the bottom of the melt using tongs, accompanied by gentle stirring to ensure thorough decomposition and gas release within the melt. Each degassing treatment lasted approximately 2 min. Upon completion of both cycles, the dross on the melt surface was skimmed off, and the melt was allowed to rest for 10 min to further eliminate inclusions and residual gases, thereby enhancing melt cleanliness. After skimming the slag, the melt was poured into a permanent steel mold preheated to 250 ± 10 °C. The cooling rate of the castings was approximately 15–25 °C/s. After demolding, the sprue and riser at the top of the ingot were excised, and specimens were sectioned from the central region of the remaining ingot.

The chemical compositions of the as-cast alloys were determined using an Olympus INNOV-X handheld X-ray fluorescence (XRF, Olympus Scientific Solutions Americas Inc., Waltham, MA, USA) spectrometer, and the measured compositions are listed in Table 1. Based on the calculations, the reaction yield ranged from 85.7% to 96.1%, indicating a relatively high in situ synthesis efficiency.

### 2.3. Heat Treatment and Thermal Exposure

The as-cast alloys obtained from melting were sectioned and subjected to a T6 heat treatment in a YYX1200-40 JINDUN (Shiyan electric furnace, Shanghai, China), with specimen dimensions of 10 mm × 10 mm × 100 mm. The specimens were heated to 540 ± 5 °C at 10 °C/min and held for 8 h. Subsequently, the specimens were rapidly transferred into water at 25 °C for quenching, with the transfer time controlled to within 10 s; circulating water was used during quenching to maintain a constant temperature. Artificial aging was conducted at 170 ± 5 °C for 4 h, with a heating rate of 10 °C/min, followed by air cooling to room temperature upon completion.

Both the T6-treated base alloy and the 4.5 wt.% Al_3_Zr/Al-Cu-Mn-Ti composite were placed in a Kington electric furnace and subjected to thermal exposure at 350 ± 5 °C for 12 h and 24 h, respectively, to investigate the influence of Al_3_Zr addition on the elevated-temperature microstructural stability of the alloys.

### 2.4. Microscopic Examination

The microstructures and elemental compositions of phases in the specimens were examined using a Phenom Pro X scanning electron microscope (SEM) equipped with an energy dispersive spectroscopy (EDS) system (Phenom-World, Eindhoven, the Netherlands). SEM observations were conducted in secondary electron (SE) imaging mode, with an accelerating voltage of 15 kV and a working distance of 8–10 mm. Metallographic specimens were ground and polished according to standard procedures, and subsequently etched using Keller’s reagent for approximately 10–15 s per cycle; the etching process was repeated twice. The specimens were immediately removed once uniform fine bubbles appeared on the surface, thoroughly rinsed with alcohol, and then dried. For microstructural characterization, no fewer than five random fields of view were selected from each specimen for observation and analysis. To quantitatively characterize the microstructural features, stereological statistical analysis of SEM images was performed using ImageJ 1.53t software. The precipitate sizes were measured using Nano Measure 1.2 software, and all reported data represent the average values obtained from over 200 independent measurements.

Phase analysis of the specimens was conducted by X-ray diffraction (XRD, Rigaku Corporation, Tokyo, Japan). Cu Kα radiation (λ = 0.15406 nm) was employed as the radiation source, with an operating voltage of 40 kV and a current of 40 mA. Data were collected over a 2θ range of 20–80°, with a step size of 0.02° and a scanning rate of 6°/min. Phase identification and indexing were performed using standard PDF cards, and the diffraction peaks were further analyzed using refinement-fitting methods.

### 2.5. Tensile Property Testing

**Figure 1:**
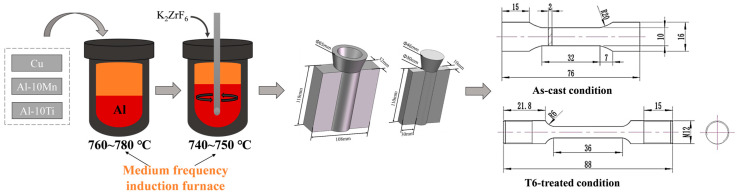
Fabrication of *x*Al_3_Zr/Al-Cu-Mn-Ti composites.

Tensile tests at room and elevated temperatures were conducted on a WDW3100 microcomputer-controlled electronic universal testing machine (Airma, Shenzhen, China). Standard flat dog-bone specimens with a gauge length of 32 mm were employed for room-temperature tensile testing. Standard cylindrical specimens with a gauge length of 36 mm were used for elevated-temperature tensile tests; all specimens were extracted from the central region of the ingots, with the machining direction aligned with the casting direction. The tensile tests were performed in accordance with GB/T 228.1–2021 (Metallic materials—Tensile testing—Part 1: Method of test at room temperature) [26] and GB/T 228.2–2015 (Metallic materials—Tensile testing—Part 2: Method of test at elevated temperature) [27]; an extensometer was employed to measure specimen deformation during testing. A resistance furnace was used to heat during elevated-temperature tensile tests at a heating rate of 10 °C/min. Upon reaching 350 °C, the specimens were held for 20 min to ensure a uniform, stable temperature distribution throughout each specimen. The temperature was controlled to within ±2 °C, and the tests were conducted in air. Subsequently, tensile loading was applied at a crosshead speed of 0.5 mm/min, and load–displacement data were recorded. A minimum of three parallel specimens were tested for each condition, and the final results are presented as mean ± standard deviation. To assess the statistical significance of differences among groups, a one-way analysis of variance (ANOVA) followed by Tukey’s honestly significant difference (HSD) post hoc test was conducted at a significance level of *p* < 0.05. In the bar charts, mean values that do not share a common lowercase letter are significantly different (*p* < 0.05), whereas those sharing at least one common letter are not statistically distinguishable. Data processing and statistical analysis were performed using Origin 2024SR1 software. The composite preparation procedure and the dimensions of the standard tensile specimens for room- and elevated-temperature tests are illustrated in Figure 1.

## 3. Results and Discussions

### 3.1. Microstructure of Al_3_Zr/Al-5Cu-0.6Mn-0.15Ti Composites in As-Cast and T6-Treated States

**Figure 2:**
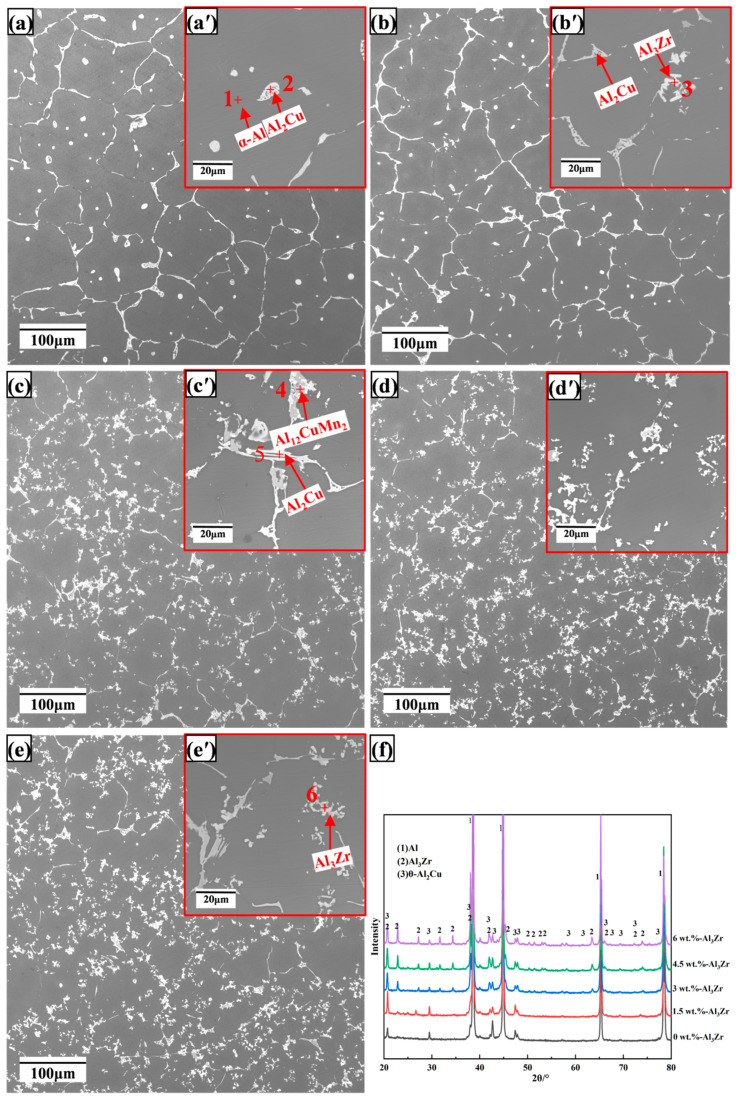
Microstructures of as-cast Al_3_Zr/Al-5Cu-0.6Mn-0.15Ti composites: (**a**) 0 wt.% Al_3_Zr; (**b**) 1.5 wt.% Al_3_Zr; (**c**) 3 wt.% Al_3_Zr; (**d**) 4.5 wt.% Al_3_Zr; (**e**) 6 wt.% Al_3_Zr; (**a′**–**e′**) magnified views of (**a**–**e**), respectively; (**f**) XRD diffraction pattern of as-cast alloy.

**Figure 3:**
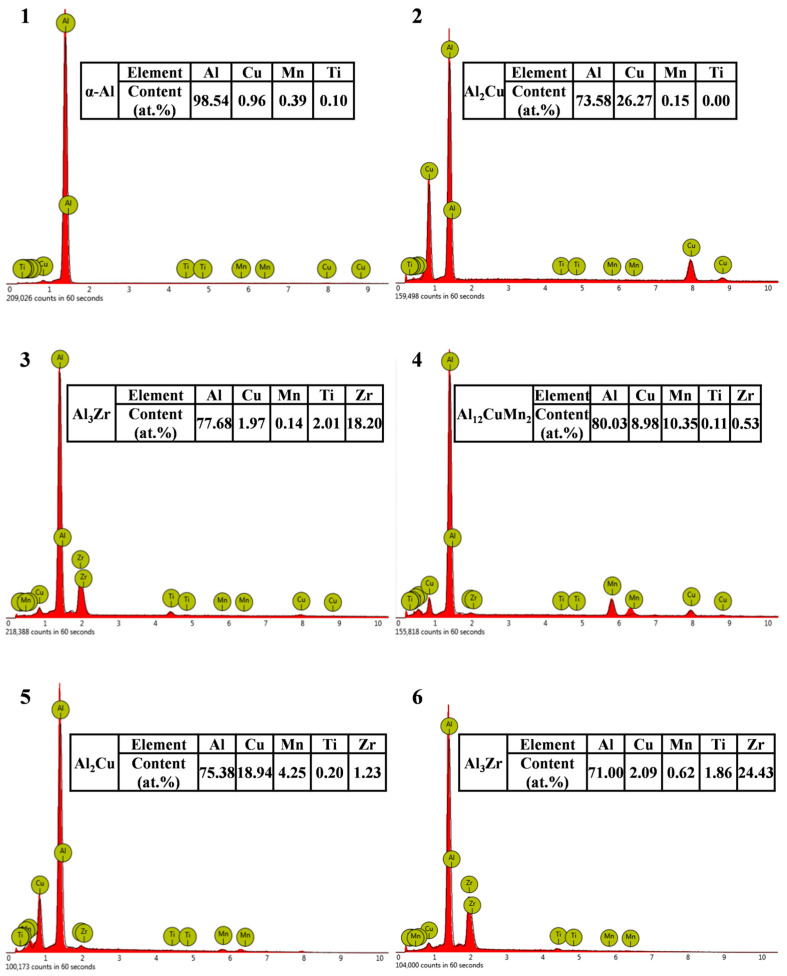
EDS analysis of points 1 to 6 in Figure 2.

The SEM micrographs and the corresponding EDS results for the as-cast *x*Al_3_Zr/Al–Cu–Mn–Ti composites are presented in Figure 2 and Figure 3, respectively. EDS and XRD analyses revealed that the as-cast microstructure primarily consists of α-Al, Al_2_Cu, Al_3_Zr, and Mn-rich phases. The dark-gray matrix corresponds to α-Al, while the bright-white network structure distributed along grain boundaries is the eutectic structure of α-Al and θ-Al_2_Cu. The Al_3_Zr phase is dispersed within the α-Al matrix and appears as blocky or rod-like particles. The grayish-white irregular blocky phases were identified as Mn-rich intermetallics. Based on the EDS analyses, in conjunction with relevant literature [28], these Mn-rich phases were identified as Al_12_CuMn_2_.

Figure 2f presents the XRD patterns of the as-cast composites. Phase identification and peak fitting analyses were performed using Jade 6.5 software, revealing three predominant phases: α-Al, θ-Al_2_Cu, and D0_23_-Al_3_Zr. These phases were indexed against the standard PDF cards #04-0787, #25-0012, and #07-0115 for α-Al, θ-Al_2_Cu, and D0_23_-Al_3_Zr, respectively. The characteristic peak intensity of D0_23_-Al_3_Zr increased progressively with increasing Al_3_Zr content. In view of the partial overlap between certain Al_3_Zr diffraction peaks and those of α-Al and θ-Al_2_Cu, peak fitting methods were employed to deconvolute the overlapping peaks. For the 4.5 wt.% Al_3_Zr composite, the fitted lattice parameters of the Al_3_Zr phase were determined to be a = b = 4.005 Å and c = 17.285 Å, with a space group of I4/mmm. These values are consistent with the lattice parameters of D0_23_-Al_3_Zr reported by Knipling et al. [29] (a = b = 4.014 Å, c = 17.321 Å). In addition, P.B. Desch et al. [30] reported that Al_3_Zr particles with a tetragonal D0_23_ structure are commonly formed in as-cast composites. Consequently, the in situ synthesized Al_3_Zr in the present work is confirmed to possess a D0_23_-type tetragonal crystal structure. Compared with the L1_2_ variant, the D0_23_-Al_3_Zr phase exhibits greater thermal stability [31] due to its resistance to coarsening and dissolution at elevated temperatures. This greater stability enables sustained dislocation pinning and suppresses grain boundary migration, thereby improving the composite’s elevated-temperature strength.

To further quantitatively evaluate the influence of Al_3_Zr addition on the as-cast microstructure, statistical analyses of the average size and area fraction of each constituent phase were conducted, and the results are summarized in Table 2. As shown in Table 2, the average grain size of α-Al progressively decreased with increasing Al_3_Zr content from 0 to 4.5 wt.%, indicating that the in situ formed Al_3_Zr particles exerted a grain-refining effect on the α-Al matrix. Concurrently, the area fraction of the Al_3_Zr phase increased. At the same time, its average size decreased, suggesting that under appropriate K_2_ZrF_6_ addition, the number of in situ formed Al_3_Zr particles continuously increased with a more uniform dispersion, which was conducive to promoting heterogeneous nucleation and grain boundary pinning. This is largely because D0_23_-Al_3_Zr and α-Al match well crystallographically, with a lattice mismatch of less than 6%. A well-defined orientation relationship exists between the two phases: [101]_Al_(11$\bar{1}$)_Al_//[1$\bar{1}$0]_Al3Zr_(114)_Al3Zr_ and [1$\bar{1}$0]_Al_(11$\bar{1}$)_Al_//[40$\bar{1}$]_Al3Zr_(114)_Al3Zr_ [32]. Hence, the in situ formed D0_23_-Al_3_Zr particles can serve as heterogeneous nucleation sites for α-Al. Based on the Edge-to-Edge model proposed by Zhang et al. [33], the relatively low lattice mismatch reduces the nucleation energy barrier, promoting the preferential formation of D0_23_-Al_3_Zr particles during solidification. These particles then provide numerous nucleation sites for α-Al, resulting in a finer as-cast grain structure. When the Al_3_Zr content was further increased to 6 wt.%, the average size of the Al_3_Zr phase increased, indicating that the Al_3_Zr particles aggregated and grew, thereby weakening the grain-refinement effect on α-Al.

Additionally, the average size of the θ-Al_2_Cu phase gradually decreased with increasing Al_3_Zr content, whereas the area fraction exhibited only a minor variation. This indicates that the θ-Al_2_Cu phase became more uniformly dispersed, and the introduction of Al_3_Zr did not significantly alter the overall content of the θ-Al_2_Cu phase, but rather primarily affected its morphology and dimensional characteristics. It is believed that the in situ reaction between K_2_ZrF_6_ and the aluminum melt consumes a portion of the aluminum matrix. Given a substantially constant total alloy mass, the relative concentration of Cu theoretically increases slightly, which favors the nucleation of the θ-Al_2_Cu phase. Meanwhile, the uniformly dispersed D0_23_-Al_3_Zr particles exert a pinning effect on grain boundary migration, thereby suppressing the growth of the eutectic θ-Al_2_Cu phase and ultimately promoting its distribution along grain boundaries in a finer, more discontinuous morphology.

**Figure 4:**
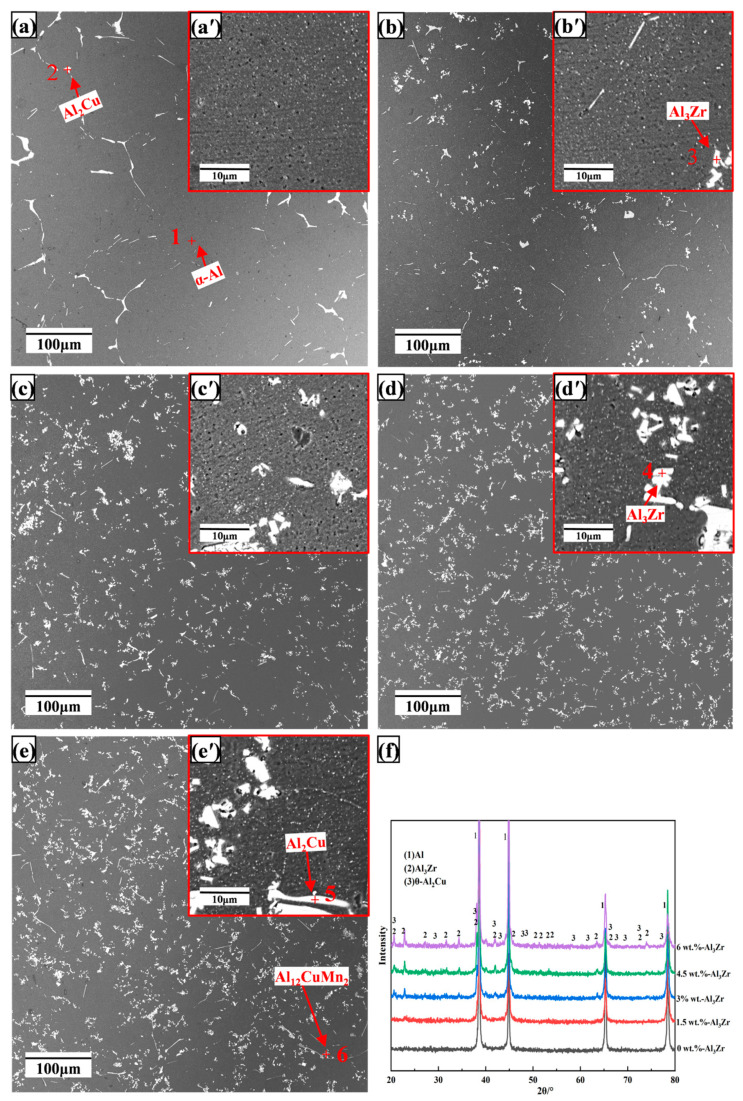
Microstructure of heat-treated Al_3_Zr/Al-5Cu-0.6Mn-0.15Ti composites: (**a**) 0 wt.% Al_3_Zr; (**b**) 1.5 wt.% Al_3_Zr; (**c**) 3 wt.% Al_3_Zr; (**d**) 4.5 wt.% Al_3_Zr; (**e**) 6 wt.% Al_3_Zr; (**a′**–**e′**) magnified views of (**a**–**e**), respectively; (**f**) XRD diffraction pattern of heat treatment alloy.

**Figure 5:**
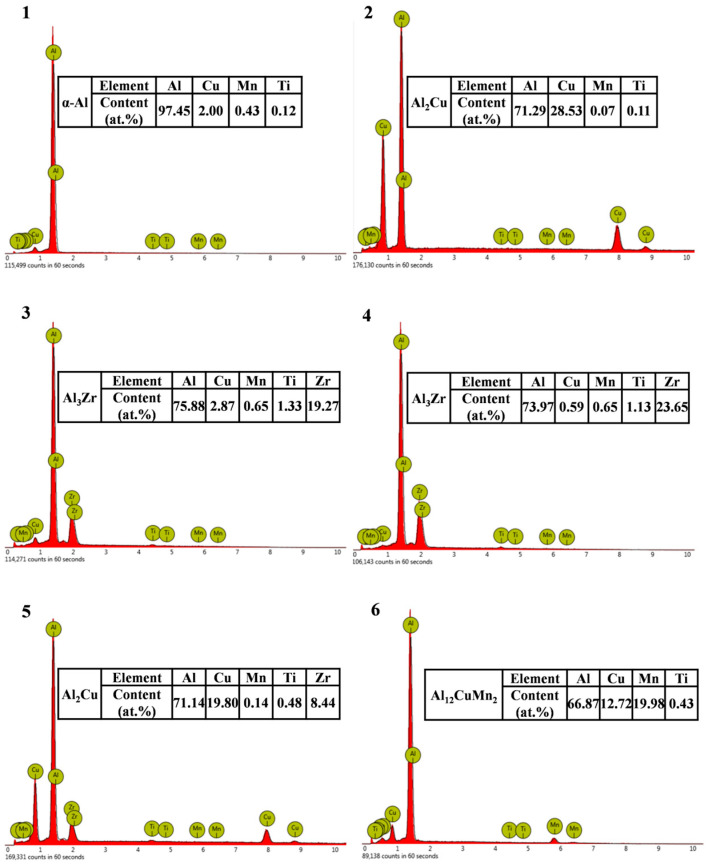
EDS analysis of points 1 to 6 in Figure 4.

Figure 4 and Figure 5 present the SEM micrographs and corresponding EDS analyses of the T6-treated *x*Al_3_Zr/Al–Cu–Mn–Ti composites. Upon T6 heat treatment, a large number of finely dispersed precipitates formed within the matrix. Given the limited spatial resolution of SEM, the present observations do not permit unambiguous discrimination among the metastable precipitate variants (GP zones, θ″, and θ′). Based on the well-established age-hardening precipitation sequence of Al-Cu alloys and the present aging condition, the dominant strengthening precipitate at this stage is expected to be the plate-like θ′-Al_2_Cu phase, and definitive identification of the precipitate type and its interfacial character would require TEM/HRTEM. On this basis, the T6-treated microstructure is taken to consist of α-Al, residual θ-Al_2_Cu, Al_12_CuMn_2_, Al_3_Zr, and fine θ′-Al_2_Cu precipitates.

To further quantitatively characterize the microstructural features of individual phases after T6 heat treatment, the average sizes and area fractions of the α-Al, θ-Al_2_Cu, and Al_3_Zr phases in the T6-treated composites were statistically analyzed, and the results are presented in Table 3. Because the thickness of the fine, plate-like θ′-Al_2_Cu precipitates in the as-aged condition lies close to the resolution limit of SEM, their thickness and area fraction were not systematically quantified as a function of Al_3_Zr content.

The α-Al grains undergo noticeable growth compared with the as-cast state, which is mainly because the high solution treatment temperature supplies enough thermal energy to drive grain boundary movement; at the same time, the eutectic Al_2_Cu network that originally pinned the as-cast grain boundaries dissolves during solution treatment, sharply lowering the resistance to boundary migration and thus facilitating grain coarsening. Compared with the alloy without Al_3_Zr addition (Figure 4a), the composite containing Al_3_Zr maintains a relatively fine grain size after heat treatment (Figure 4b–e). This can be explained by the dispersed Al_3_Zr particles, which, at the solution temperature, are located along grain boundaries or within grains, where they strongly pin grain boundary migration and consequently inhibit grain coarsening during heat treatment. Therefore, although grain growth occurs after heat treatment, a higher Al_3_Zr content leads to stronger grain-boundary pinning, thereby more effectively suppressing grain coarsening. However, the magnitude of grain growth in the composites was considerably lower than that in the base alloy. As the Al_3_Zr content increased from 0 to 6 wt.%, the average α-Al grain size progressively decreased, indicating that the Al_3_Zr particles could continuously exert a grain boundary pinning effect during solution treatment, thereby effectively suppressing grain boundary migration and grain coarsening. Furthermore, the area fraction of the Al_3_Zr phase progressively increased with increasing Al_3_Zr addition. Compared with the as-cast condition, the Al_3_Zr particles exhibited slight coarsening with a reduced number density, suggesting that a fraction of the finer Al_3_Zr particles dissolved or coarsened during solution treatment. Nevertheless, the overall variation in the Al_3_Zr particles was relatively minor, demonstrating reasonably favorable thermal stability.

After T6 heat treatment, the average size of the residual θ-Al_2_Cu phase decreased relative to the as-cast condition, accompanied by a decrease in its area fraction. This indicates that a substantial amount of the eutectic θ-Al_2_Cu phase dissolved during solution treatment, providing solute atoms for the precipitation of θ′-Al_2_Cu during aging, with only a small amount of undissolved residual θ-Al_2_Cu phase retained at grain boundaries or within grains. As shown in Figure 4(b′–e′), fine and uniformly distributed θ′-Al_2_Cu precipitates were formed within the composite matrix upon heat treatment. Within the resolution of SEM, the incorporation of Al_3_Zr produced no marked change in the apparent distribution of the θ′-Al_2_Cu phase relative to the alloy without Al_3_Zr addition. In Al-Cu alloys, the metastable strengthening precipitates are well known to evolve through the sequence supersaturated solid solution → GP zones → θ″ → θ′ [34]. The precipitation process is mainly driven by the supersaturated Cu atoms retained in the α-Al matrix after quenching. On the other hand, the nucleation and growth kinetics of the θ′-Al_2_Cu phase are primarily controlled by the diffusivity of Cu in the Al matrix. Al_3_Zr is a thermally stable dispersoid phase that precipitates during solidification and is resistant to dissolution at solution treatment temperatures, whereas the θ′ phase nucleates and grows during aging. These two phases form at quite different temperatures. Al_3_Zr completes its precipitation ahead of the θ′ phase. During the aging process, the Al_3_Zr particles neither serve as heterogeneous nucleation sites for the θ′ phase, nor do they absorb or supply Cu atoms. Consequently, the addition of Al_3_Zr exerts only a minor influence on the heterogeneous nucleation of θ′-Al_2_Cu and on the overall partitioning of Cu [35]. The present SEM-scale results therefore suggest that, to a first approximation, the strengthening contributions of the Al_3_Zr particles and the θ′-Al_2_Cu precipitates can be treated as largely independent under the present processing conditions.

### 3.2. Room-Temperature Mechanical Properties

**Figure 6:**
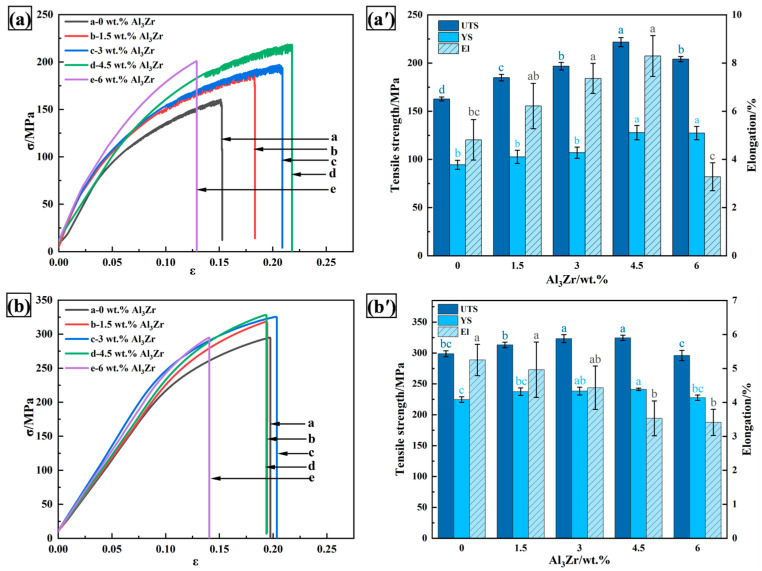
Room-temperature tensile stress–strain curves and bar charts summarizing tensile properties for *x*Al_3_Zr/Al-5Cu-0.6Mn-0.15Ti alloys: (**a**) stress–strain curves in the as-cast condition; (**a′**) corresponding tensile data in the as-cast state; (**b**) stress–strain curves in the T6-treated condition; (**b′**) corresponding tensile data in the T6-treated state.

Figure 6a,b present the room-temperature tensile stress–strain curves of the *x*Al_3_Zr/Al-5Cu-0.6Mn-0.15Ti composites under as-cast and T6-treated conditions, respectively. To ensure the reliability of the test results, a minimum of three parallel tensile specimens were tested for each experimental condition, and the representative stress–strain curves are shown in the figures. Figure 6(a′,b′) illustrate the corresponding tensile data in the form of bar charts.

According to Figure 6(a′), the room-temperature tensile strength and yield strength of the as-cast composites rise with increasing Al_3_Zr content, reach a peak, and then gradually decline. The composite with 4.5 wt.% Al_3_Zr achieved an average tensile strength of 221.72 MPa, representing a 36.24% improvement over the base alloy, and an average yield strength of 127.80 MPa, corresponding to a 35.41% enhancement; as indicated by the different letter groups in Figure 6(a′), both improvements relative to the base alloy are statistically significant (*p* < 0.05). The elongation followed a similar trend of an initial increase and subsequent decrease, rising from 4.81% to 8.29% over the Al_3_Zr content range of 0.0–4.5 wt.% and then dropping significantly to 3.28% at 6 wt.%. Experimental findings indicate that incorporating Al_3_Zr particles substantially enhances the overall mechanical properties of the base alloy. This improvement stems largely from the inherent hardness, mechanical strength, and thermal stability of Al_3_Zr particles, which strengthen the matrix primarily through grain refinement, load transfer, and dispersion strengthening. Figure 2 illustrates that fine Al_3_Zr particles uniformly dispersed in the matrix (e.g., at 4.5 wt.%) obstruct dislocation glide by pinning grain boundaries. In parallel, these particles facilitate matrix grain refinement. These factors work in concert to simultaneously increase tensile strength and elongation, building on the already relatively high room-temperature strength of the base alloy. Once the Al_3_Zr content reaches 6 wt.%, however, excessive particle clustering amplifies interfacial stress concentrations, a finding consistent with the cleavage fracture morphology observed on the fracture surfaces. These stress concentrations promote crack propagation, which, in turn, impairs the mechanical performance.

As shown in Figure 6(b′), the room-temperature tensile strength and yield strength of the T6-treated composites both exhibited an initial increase followed by a decrease with increasing Al_3_Zr content. The composite with 4.5 wt.% Al_3_Zr achieved average tensile strength and yield strength of 324.44 MPa and 241.16 MPa, respectively, corresponding to improvements of 8.56% and 7.38% over the base alloy; the post hoc test confirms that these increases relative to the base alloy are statistically significant. The elongation showed an overall decreasing trend, with the 4.5 wt.% and 6 wt.% composites exhibiting significantly lower elongation than the base alloy. Following treatment, the particle edges rounded and gradually became elliptical, thereby reducing interfacial stress concentrations. Meanwhile, the heat treatment induced the precipitation of fine θ′-Al_2_Cu particles with great thermal stability. Such refinement intensified the dispersion-strengthening effect, thereby increasing the tensile properties of the composites. The combined effect of these two factors enabled the composites to retain high strength, albeit at the expense of partial ductility.

To further analyze the effect of Al_3_Zr content on the alloy’s mechanical properties, a quantitative assessment of its strengthening mechanisms was performed. The primary sources of strengthening include grain boundary strengthening (∆σ_gb_), dislocation strengthening (∆σ_d_), precipitation strengthening (∆σ_p_) and load-bearing mechanism (∆σ_load_), which can be expressed by the following formula [11,36]:

(2) $$\begin{array}{c} \sigma_{\mathrm{YS}}\;=\;{\Delta \sigma }_{\mathrm{gb}}\;+\;\Delta \sigma_{d}\;+\;\Delta \sigma_{p}\;+\;∆\sigma_{load} \end{array}$$

The grain-boundary contribution follows the Hall–Petch relationship [37]:

(3) $$\begin{array}{c} \Delta \sigma_{\mathrm{gb}}\;={\;\sigma_{0}\;+\;k}_{y}d^{-1/2} \end{array}$$

where σ_0_ is the lattice friction stress, k_y_ is the Hall-Petch constant and d is the average grain size. This relationship indicates that a reduction in grain size increases in ∆σ_gb_, as finer grains provide more grain boundary area to impede dislocation motion. In the present work, the addition of Al_3_Zr particles refines the α-Al grains from 74.50 μm (in the base alloy) to 46.66 μm, thereby contributing significantly to the overall strengthening.

The dislocation contribution originates from the geometrically necessary dislocations generated at the Al_3_Zr/α-Al interface during cooling, owing to the mismatch in the coefficient of thermal expansion (CTE) between the D0_23_-Al_3_Zr particles and the α-Al matrix. The resulting dislocation density and the corresponding strengthening are given by the geometrically necessary dislocation density and the Taylor dislocation-strengthening relation [38,39]:

(4) $$\begin{array}{c} \rho \;=\;12\Delta \alpha \;\cdot \;\Delta T\;\cdot {\;V}_{p}/[b\;\cdot \;d_{p}\;\cdot \;(1-V_{p})] \end{array}$$

(5) $$\begin{array}{c} \Delta \sigma_{d}=M\alpha Gb\rho^{1/2} \end{array}$$

where Δα is the CTE mismatch, ΔT the cooling range from the aging temperature to room temperature, V_p_ and d_p_ the volume fraction and mean diameter of the particles, M the Taylor factor, α a constant, G the shear modulus and b the Burgers vector.

The precipitation contribution from the plate-like θ′-Al_2_Cu phase is described by the Orowan expression for disk-shaped precipitates [40]:

(6) $$\begin{array}{c} \sigma_{p}=\frac{\mathrm{MGb}}{2\pi \sqrt{1-v}}\frac{1}{0.931\sqrt{\frac{0.306\pi \mathrm{dt}}{f_{v}f_{\mathrm{ma}}}}}\ln\left(\frac{0.981\sqrt{\mathrm{dt}}}{b}\right) \end{array}$$

where ν is Poisson’s ratio, f_ma_ the maximum absolute volume fraction of the precipitate, and d, t, and f_v_ the mean diameter, thickness, and volume fraction of θ′. Because θ′ nucleates and grows during aging independently of the pre-existing Al_3_Zr particles, Δσ_p_ is governed by the aging response of the Cu-bearing matrix and is essentially insensitive to the Al_3_Zr addition. However, because these θ′ dimensions lie close to the SEM resolution limit and were not quantified in the present work, Δσ_p_ is not evaluated numerically here.

∆σ_load_ is the load-transfer contribution from the particles, which can be estimated by the Shear-Lag model [41]:

(7) $$\begin{array}{c} \Delta \sigma_{\mathrm{load}}\;=\;0.5V_{p}\sigma_{m} \end{array}$$

where V_p_ is the volume fraction of the particles and σ_m_ is the yield strength of the unreinforced matrix. This model describes the capacity of hard particles to bear a portion of the applied load through effective interfacial stress transfer. As V_p_ increases, the load-transfer contribution becomes more pronounced. In the present work, the Al_3_Zr area fraction increases from 0 to 4.57%, promoting a substantial load-transfer strengthening effect.

Based on the above framework, the strengthening contributions fall into two largely independent groups: the Al_3_Zr-related terms (Δσ_gb_, Δσ_load_, and Δσ_d_), which all increase with Al_3_Zr content, and the matrix-controlled precipitation term Δσ_p_ discussed above. As the Al_3_Zr content increases from 0 to 4.5 wt.%, the progressive grain refinement and the rising particle volume fraction simultaneously enhance ∆σ_gb_, ∆σ_load_, and ∆σ_d_, so that the overall strength increases and peaks at 4.5 wt.% Al_3_Zr. When the content is further increased to 6 wt.%, particle agglomeration and coarsening reduce dispersion uniformity and introduce interfacial stress concentrations; the consequent reduction in effective load transfer, together with the promotion of premature crack initiation, outweighs the marginal grain-refinement benefit and leads to a decrease in strength.

**Figure 7:**
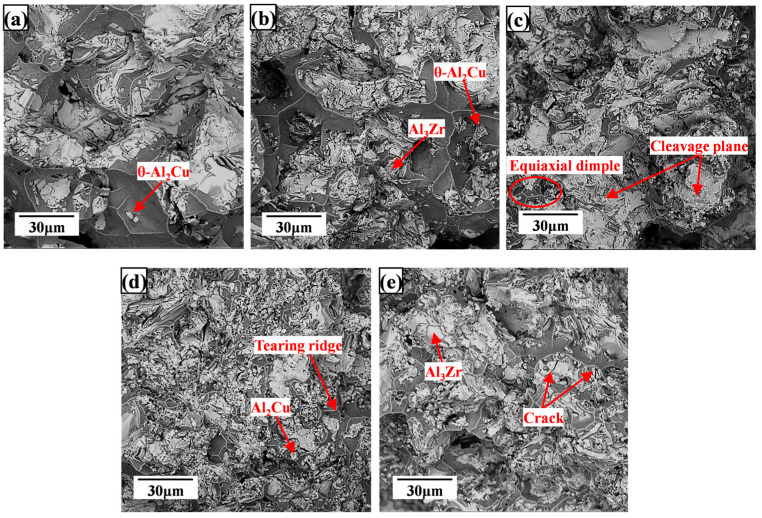
Room temperature tensile fracture surface of as-cast *x*Al_3_Zr/Al-5Cu-0.6Mn-0.15Ti alloy: (**a**) 0 wt.% Al_3_Zr; (**b**) 1.5 wt.%; (**c**) 3 wt.% Al_3_Zr; (**d**) 4.5 wt.% Al_3_Zr; (**e**) 6 wt.% Al_3_Zr.

Figure 7 presents the room-temperature tensile fracture morphologies for composites prepared under as-cast conditions. The fracture surface of the as-cast alloy is characterized by dominant cleavage facets and a sparse distribution of dimples, suggesting a combined ductile-brittle fracture mechanism. After Al_3_Zr is introduced, θ-Al_2_Cu and Al_3_Zr phases become the principal constituents of the as-cast composite fracture surfaces. The Al_3_Zr particles are not surrounded by dimples but instead lie on flat fracture facets, indicating cleavage fracture. The fracture surface also contains cleavage steps, tear ridges, and a limited number of equiaxed dimples, indicating that localized plastic deformation occurred during crack propagation. During tensile deformation, the brittle θ-Al_2_Cu and Al_3_Zr phases exhibit a large elastic modulus mismatch with the matrix, resulting in interfacial stress concentration and making them prone to act as crack initiation sites. Microcracks first form at these locations, then propagate along second-phase particles or particle/matrix interfaces, and gradually coalesce into main cracks, ultimately leading to fracture failure. This fracture feature is consistent with the stress–strain behavior shown in Figure 6a, where the material rapidly loses stability and fractures after reaching peak strength, indicating limited plasticity and a fracture process dominated by brittle mechanisms. As the Al_3_Zr particle content increases, the tear ridges gradually shrink in size, while the dimples become finer and display a more uniform distribution. This enhancement is primarily due to the grain-refinement strengthening imparted by Al_3_Zr particles to the α-Al matrix, thereby improving the tensile properties of the composites. Once the Al_3_Zr content reaches 6 wt.%, particle agglomeration facilitates rapid crack propagation through the clustered regions, thereby accelerating fracture and degrading room-temperature tensile performance.

**Figure 8:**
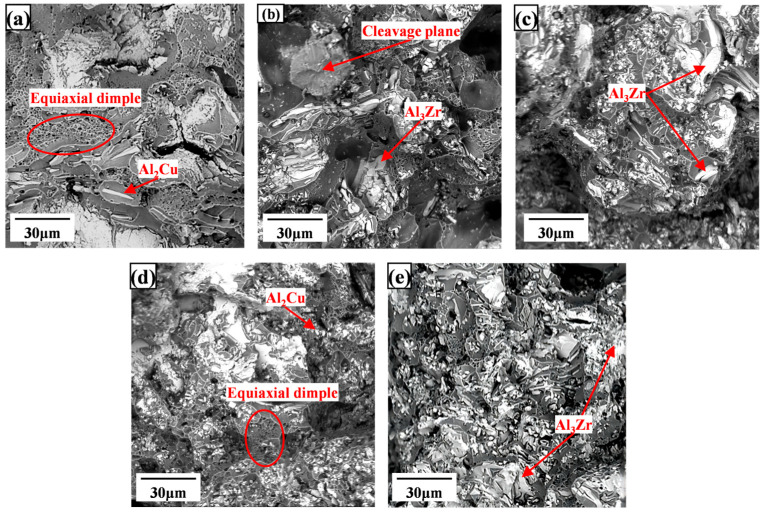
Room temperature tensile fracture surface of heat-treated *x*Al_3_Zr/Al-5Cu-0.6Mn-0.15Ti alloy: (**a**) 0 wt.% Al_3_Zr; (**b**) 1.5 wt.% Al_3_Zr; (**c**) 3 wt.% Al_3_Zr; (**d**) 4.5 wt.% Al_3_Zr; (**e**) 6 wt.% Al_3_Zr.

Figure 8 shows the room-temperature tensile fracture morphology of the heat-treated composites. The room-temperature tensile fracture surface of the T6-treated composite exhibits equiaxed dimples, cleavage facets, and tear ridges, indicating a mixed ductile-brittle fracture mode that is predominantly brittle. After T6 heat treatment, the fracture surface shows fewer cleavage facets and more dimples than in the as-cast condition. Compared with the fracture surface of the as-cast base alloy, that of the T6-treated base alloy exhibited fewer cleavage facets and an increased number of dimples, with fine θ′-Al_2_Cu precipitates observed at the dimple bottoms. Upon the addition of Al_3_Zr, the fracture surface exhibits a higher proportion of cleavage facets than in the base alloy. As the Al_3_Zr content increases, the tear ridges become finer, and the tendency toward brittle fracture grows. This is mainly attributed to the pinning action of the more numerous, dispersed Al_3_Zr particles and θ′-Al_2_Cu precipitates on dislocation motion, which impede dislocation glide and consequently reduce the capacity for plastic deformation. At an Al_3_Zr content of 6 wt.%, particle agglomeration occurs; microvoids nucleate and quickly coalesce into microcracks, producing large areas of cleavage facets on the fracture surface typical of brittle fracture, along with a decline in mechanical properties.

### 3.3. Elevated-Temperature Mechanical Properties

**Figure 9:**
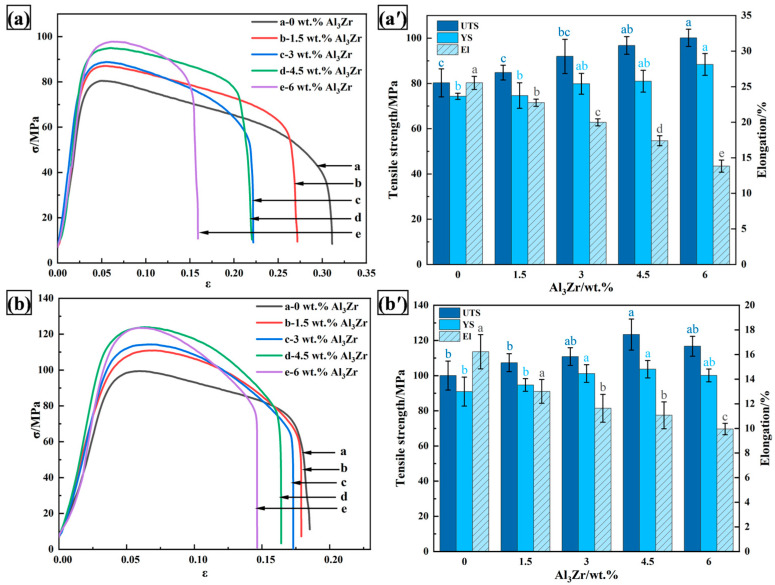
Elevated-temperature tensile stress–strain curves and bar charts summarizing tensile properties for *x*Al_3_Zr/Al-5Cu-0.6Mn-0.15Ti alloys: (**a**) stress–strain curves in the as-cast condition; (**a′**) corresponding tensile data in the as-cast state; (**b**) stress–strain curves in the T6-treated condition; (**b′**) corresponding tensile data in the T6-treated state.

Figure 9 shows the stress–strain curves and tensile-property bar charts for *x*Al_3_Zr/Al-Cu-Mn-Ti composites tested at 350 °C, covering both as-cast and T6-treated conditions. To ensure the reliability of the test results, a minimum of three parallel tensile specimens were tested for each experimental condition, and the representative stress–strain curves are shown in the figures.

**Figure 10:**
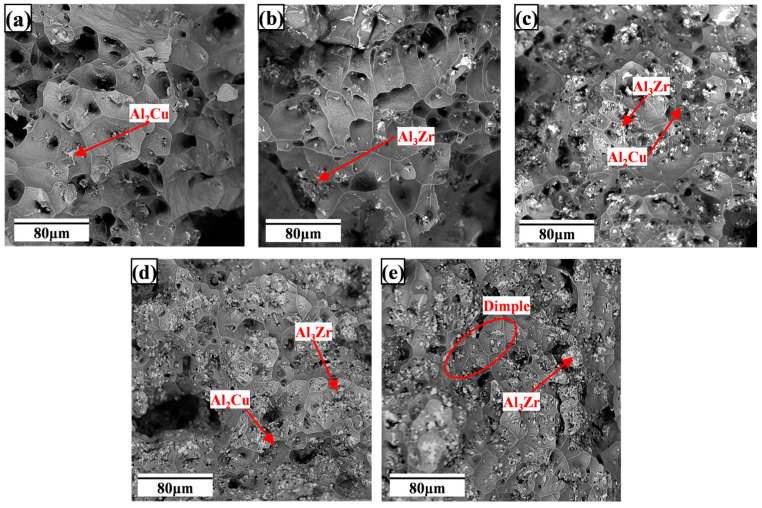
Elevated-temperature tensile fracture surface of as-cast *x*Al_3_Zr/Al-5Cu-0.6Mn-0.15Ti alloy: (**a**) 0 wt.% Al_3_Zr; (**b**) 1.5 wt.% Al_3_Zr; (**c**) 3 wt.% Al_3_Zr; (**d**) 4.5 wt.% Al_3_Zr; (**e**) 6 wt.% Al_3_Zr.

As shown in Figure 9(a′), as the Al_3_Zr content rises, both the tensile strength and yield strength of as-cast composites increase, yet their elongation gradually declines. The as-cast alloy with 6 wt.% Al_3_Zr achieved the highest average tensile strength of 100.13 MPa, representing an improvement of approximately 24.77% over the base alloy, and an average yield strength of 88.38 MPa, corresponding to an enhancement of 18.97%; both improvements are statistically significant relative to the base alloy (*p* < 0.05). In contrast, the progressive reduction in elongation with increasing Al_3_Zr content is significant at every level. The variation in elongation at elevated temperature differed from that observed at room temperature for the as-cast condition. Based on the fracture surface analysis in Figure 10, it is inferred that at 350 °C, the matrix underwent significant softening and dynamic recovery, the activation energy for grain boundary migration decreased, and grain coarsening occurred. These changes led to a decrease in grain boundary density and a deterioration in the capacity for deformation coordination. Concurrently, the deformation mechanism transitioned from dislocation slip-dominated at room temperature to grain boundary sliding-dominated at elevated temperature. The pinning effect of Al_3_Zr particles on grain boundaries impeded coordinated deformation, and the particle/matrix interfaces became preferential sites for microvoid nucleation. With increasing Al_3_Zr content, the particle distribution became more concentrated, the number of microvoid nucleation sites increased, resulting in finer and shallower dimples, and the capacity for plastic deformation progressively deteriorated.

As shown in Figure 9(b′), after T6 heat treatment, the average tensile and yield strengths of the T6-treated alloys at elevated temperature both initially increased, then decreased with increasing Al_3_Zr content at 4.5 wt.% Al_3_Zr, the average tensile strength reached 123.38 MPa, representing a 23.31% improvement over the base alloy, and the average yield strength attained 103.67 MPa, corresponding to a 14.02% enhancement; both improvements relative to the base alloy are statistically significant (*p* < 0.05). The elongation showed a decreasing trend, with the 6 wt.% composite exhibiting the lowest, significantly reduced elongation. Compared with the as-cast condition, the strength of all T6-treated alloys was significantly enhanced, which was primarily attributed to the dispersion strengthening effect of the θ′-Al_2_Cu precipitates.

A comparison of the elevated-temperature tensile results between the as-cast and T6-treated composites revealed that the optimal Al_3_Zr contents for these two conditions were not identical. The as-cast composites reached their maximum tensile and yield strengths at 6 wt.% Al_3_Zr under 350 °C, whereas the T6-treated composites attained their optimal elevated-temperature properties at 4.5 wt.% Al_3_Zr. This was attributed to the significant softening and dynamic recovery of the matrix during elevated-temperature deformation of the as-cast materials, which mitigated the localized stress concentration induced by Al_3_Zr particle agglomeration. Meanwhile, the higher volume fraction of Al_3_Zr particles could continuously bear the applied load and impede dislocation motion, thereby rendering the 6 wt.% Al_3_Zr composite superior in elevated-temperature strength. In contrast, the strengthening sources of the T6-treated materials comprised not only the Al_3_Zr particles but also the numerous precipitated θ′-Al_2_Cu phases. When the Al_3_Zr content was increased to 6 wt.%, particle coarsening and agglomeration reduced the dispersion uniformity of the reinforcement phases and weakened the combined strengthening effect between the θ′-Al_2_Cu precipitates and the Al_3_Zr particles, resulting in a deterioration of elevated-temperature strength.

As analyzed in Section 3.2, the D0_23_-Al_3_Zr particles strengthen the composite mainly through load transfer and dislocation obstruction, both of which rely on the strong, well-bonded particle/matrix interface. Owing to their high thermal stability, the particles retain this strengthening capacity at 350 °C, at which the matrix itself has softened substantially. Under tensile loading at 350 °C, dynamic recovery of the matrix alleviates interfacial stress concentration and retards microcrack initiation, improving plastic deformability.

At 350 °C, first, the geometrically necessary dislocations generated by the Al_3_Zr/α-Al thermal-expansion mismatch are largely removed by dynamic recovery, so Δσ_d_ no longer provides a sustained contribution. Second, because deformation becomes grain-boundary-sliding-controlled at this temperature, the Hall–Petch term ceases to act as a net strengthening contribution. The strengthening retained from the Al_3_Zr addition is therefore dominated by load transfer and by Orowan/dispersion strengthening from the thermally stable particles. More importantly, in the T6-treated condition, the finely dispersed θ′ precipitates undergo rapid coarsening at 350 °C, leading to a progressive loss of their strengthening capability. This thermally induced degradation of the θ′ precipitates competes with the sustained strengthening provided by the thermally stable Al_3_Zr particles. It is this competition that governs the compositional dependence of the elevated-temperature tensile properties. For the T6-treated composites, the optimum at 4.5 wt.% Al_3_Zr arises mainly because the Al_3_Zr particles are then most uniformly dispersed and deliver the highest load-transfer and dispersion-strengthening efficiency. At 6 wt.%, particle agglomeration reduces the effective load-transfer area, while the associated stress concentrations accelerate localized damage, lowering the tensile strength below that of the 4.5 wt.% composite. Under as-cast conditions, the absence of θ′ precipitates means that there is no metastable strengthening phase to coarsen, and therefore no thermally induced degradation of dispersion strengthening to compete with. The Al_3_Zr particles constitute virtually the sole elevated-temperature strengthening source. In this case, as long as the particle dispersion remains reasonably uniform, a higher Al_3_Zr content provides a higher volume fraction of load-bearing particles, yielding greater load-transfer strengthening. Although particle agglomeration occurs at 6 wt.%, substantial matrix softening and dynamic recovery at 350 °C partially relax the stress concentrations associated with agglomerated clusters. Consequently, the 6 wt.% as-cast composite, despite some agglomeration, still outperforms the lower-content counterparts, achieving the highest tensile strength among all as-cast compositions.

Figure 10 presents the fracture surface morphologies of the as-cast materials after tensile testing at 350 °C. In the base alloy without Al_3_Zr, the fracture surfaces exhibit large, deep dimples, indicating significant plastic deformation before failure. The occurrence of this phenomenon was attributed to two factors: on the one hand, dislocation motion was significantly activated at 350 °C; on the other hand, dynamic recovery promoted dislocation rearrangement, thereby weakening the work-hardening effect and enabling the material to accumulate uniform plastic deformation before fracture. After the Al_3_Zr reinforcing phase is incorporated, the fracture surface appearance of the composite material changes noticeably compared with that of the base alloy. The centers of most dimples contain hard Al_3_Zr particles and some Al_2_Cu, and these dimples are considerably smaller and shallower than those observed in the base alloy. This suggests that stress concentrations develop at the Al_3_Zr/matrix interfaces during tensile deformation due to differences in the elastic modulus and thermal expansion coefficient, and that these regions serve as preferential sites for microcrack initiation [42]. With increasing Al_3_Zr content, the particle distribution becomes more concentrated, resulting in a marked increase in microvoid nucleation. These adjacent microvoids coalesce before full growth, ultimately producing finer-morphology dimples with reduced depth at elevated temperatures.

**Figure 11:**
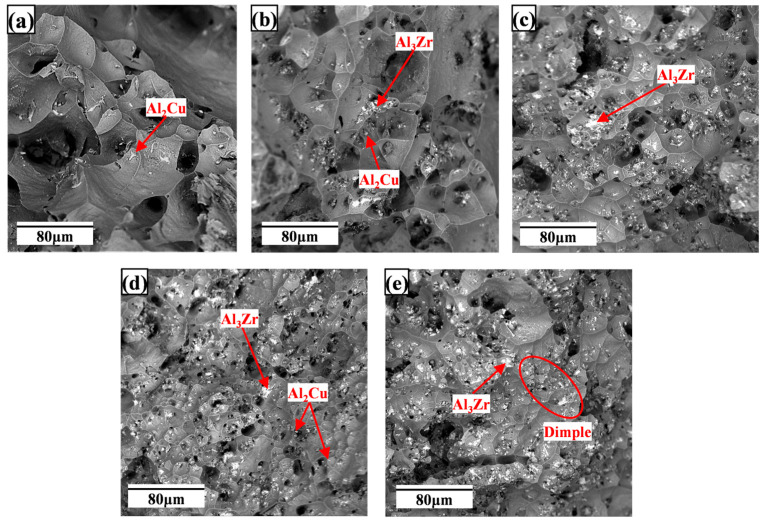
Elevated-temperature tensile fracture surface of heat-treated *x*Al_3_Zr/Al-5Cu-0.6Mn-0.15Ti alloy: (**a**) 0 wt.% Al_3_Zr; (**b**) 1.5 wt.% Al_3_Zr; (**c**) 3 wt.% Al_3_Zr; (**d**) 4.5 wt.% Al_3_Zr; (**e**) 6 wt.% Al_3_Zr.

Figure 11 presents the fracture surface morphologies of the T6-treated materials after tensile testing at 350 °C. These fracture surfaces exhibited an evolutionary trend analogous to that of the as-cast condition. Compared with the as-cast alloys, the dimples on the fracture surfaces of the T6-treated alloys were generally finer. This was primarily attributed to the pinning effect of θ′-Al_2_Cu precipitates on dislocations, which restricted the capacity for plastic deformation. The composite with 4.5 wt.% Al_3_Zr exhibited the most uniform and fine dimple distribution, which corresponded to its optimal elevated-temperature tensile properties.

The T6-treated composite with 4.5 wt.% Al_3_Zr exhibited a tensile strength of 123.38 MPa at 350 °C. A comparison with the elevated-temperature tensile strengths of similar heat-resistant aluminum alloys reported in the recent literature, as summarized in Table 4, indicates that this composite exhibits comparable mechanical properties at 350 °C, highlighting its potential applicability in elevated-temperature service environments.

### 3.4. Thermal Exposure Test

**Figure 12:**
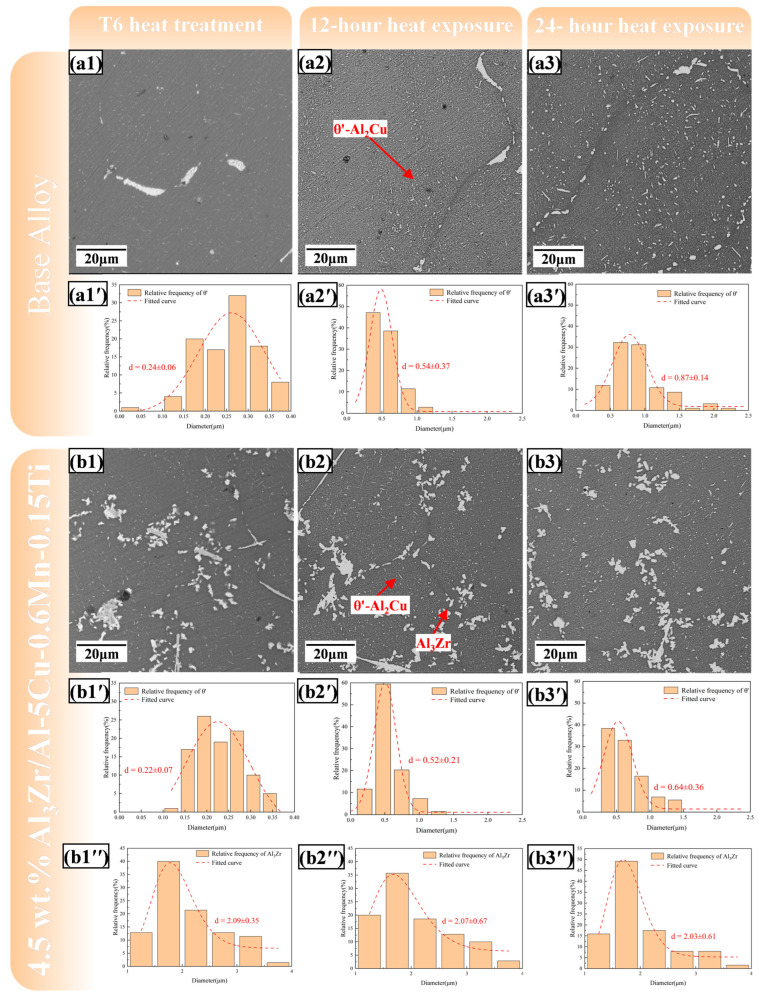
Microstructural evolution of T6-treated base alloys and 4.5 wt.% Al_3_Zr composites during thermal exposure at 350 °C: (**a1**–**a3**) SEM images of the base alloy; (**a1′**–**a3′**) Particle size distribution of θ′-Al_2_Cu; (**b1**–**b3**) SEM images of the 4.5 wt.% Al_3_Zr composite; (**b1′**–**b3′**) Particle size distribution of θ′-Al_2_Cu; (**b1″**–**b3″**) Particle size distribution of Al_3_Zr.

To examine the effect of Al_3_Zr on the precipitation behavior and elevated-temperature stability of the θ′-Al_2_Cu phase, heat-treated samples of the Al-Cu-Mn-Ti base alloy and the 4.5 wt.% Al_3_Zr/Al-Cu-Mn-Ti composite were held at 350 °C for 12–24 h; the microstructures obtained are presented in Figure 12(a1–a3, b1–b3).

To quantitatively characterize the coarsening behavior of the θ′-Al_2_Cu and Al_3_Zr phases during thermal exposure at 350 °C, statistical analyses of the diameters of the strengthening phases in the thermally exposed specimens were conducted. Figure 12(a1′–a3′,b1′–b3′) presents the particle size distributions of the θ′-Al_2_Cu phase. The θ′-Al_2_Cu phase in all alloys coarsened as the thermal exposure time was prolonged from 12 h to 24 h. After 24 h of thermal exposure, the peak of the particle size distribution in the base alloy shifted toward larger particle sizes, accompanied by a noticeable broadening of the distribution, indicating significant coarsening of the precipitates. In contrast, although the particle size distribution of the θ′ phase in the Al_3_Zr-containing composite likewise shifted toward larger sizes, the magnitude of the shift was smaller and the distribution remained more concentrated in the finer size range. This indicates that θ′ coarsening was less pronounced in the composite.

The SEM-scale particle-size distributions in Figure 12 show that the θ′ precipitates coarsen with increasing exposure time, and that this coarsening is less pronounced in the Al_3_Zr-containing composite than in the base alloy; these size trends are the direct experimental observation, whereas the underlying mechanism is interpreted below with reference to established coarsening theory. The temperature of 350 °C lies within the severe over-aging range for Al-Cu alloys, well above the thermal stability threshold of the metastable θ′-Al_2_Cu phase, so that Cu diffusion is readily activated. The precipitate population is expected to undergo Ostwald ripening: following the Gibbs–Thomson relation, smaller precipitates with higher interfacial curvature dissolve preferentially, whereas solute redeposits on larger ones, reducing the total interfacial free energy and producing the observed shift in the distribution toward larger sizes. Prolonged exposure in this regime is also expected, based on prior studies of Al-Cu alloys [51], to be accompanied by progressive loss of θ′/matrix coherency and eventual transformation toward the equilibrium θ-Al_2_Cu phase. The combined effect is a gradual decline in the elevated-temperature strengthening contribution of the precipitates.

It is important to distinguish the experimentally observed reduction in θ′ coarsening from its underlying mechanism. Coarsening of θ′-Al_2_Cu at 350 °C is governed by long-range Cu diffusion through the α-Al matrix and follows Lifshitz–Slyozov–Wagner (LSW) kinetics [52], for which the rate constant scales (K) with the product of the interfacial energy (γ), the Cu lattice diffusivity (D), and the equilibrium Cu solubility (C_e_):

(8) $$\begin{array}{c} K\;\propto \;\gamma \cdot \;D\cdot {\;C}_{e} \end{array}$$

At the exposure temperature of 350 °C, the equilibrium Cu solubility (C_e_) can be regarded as essentially fixed for a given matrix composition. Consequently, any reduction in the coarsening rate constant K for the composite relative to the base alloy must arise from a decrease in the interfacial energy (γ), the Cu lattice diffusivity (D), or both. Two factors may plausibly contribute to such a reduction. First, a small fraction of Zr retained in supersaturated solid solution after solution treatment may co-segregate with Mn to the semi-coherent θ′/α-Al interface during exposure, lowering γ and reducing ledge mobility; such an interfacial-segregation route is well documented for thermally stable θ′ in Al-Cu-Mn-Zr alloys [53]. Second, the dense, homogeneously distributed Al_3_Zr particles, together with the associated finer, more uniform matrix grain structure, may locally perturb the Cu concentration field that drives Ostwald ripening, thereby reducing the local diffusivity (D) available for coarsening. The combined effect of these mechanisms is a net reduction in the product γ·D·C_e_, which translates into a lower coarsening rate constant K for the Al_3_Zr-containing composite compared to the matrix alloy. Consequently, the θ′ precipitates in the composite exhibit less pronounced coarsening after prolonged thermal exposure. We emphasize that the factors are hypotheses consistent with, but not proven by, the present SEM/EDS data; their confirmation requires interface-resolved characterization such as atom-probe tomography or high-resolution TEM of the θ′/α-Al interface, which is beyond the scope of the present study and is identified as a direction for future work. Accordingly, throughout this work, the enhanced thermal stability of the composite is attributed primarily to the proven stability of the Al_3_Zr particle strengthening, with the reduced θ′ coarsening treated as a contributing rather than the controlling factor.

**Figure 13:**
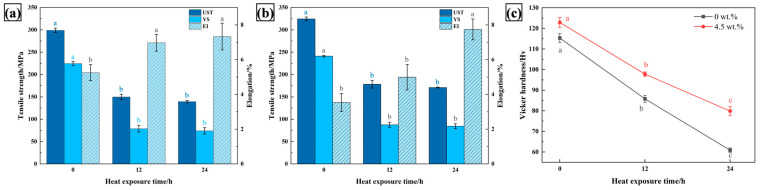
Room-temperature mechanical properties of the base alloy and the 4.5 wt.% Al_3_Zr composite after thermal exposure at 350 °C: (**a**) Bar chart of tensile data for the base alloy; (**b**) Bar chart of tensile data for the 4.5 wt.% Al_3_Zr composite; (**c**) Changes in microhardness.

To further verify the influence of Al_3_Zr on the elevated-temperature microstructural stability of the composites, room-temperature tensile tests and microhardness measurements were conducted on the base alloy and the 4.5 wt.% Al_3_Zr composite after thermal exposure at 350 °C, and the results are presented in Figure 13. As shown in Figure 13a,b, the tensile strength and yield strength of both materials decreased continuously with increasing thermal exposure time from 0 to 24 h. In contrast, the elongation progressively increased, indicating significant overaging softening. For both materials, the strengths after 12 h and 24 h are significantly lower than those in the unexposed (0 h) state (*p* < 0.05); however, the strength values at 12 h and 24 h share a common letter group, indicating that no statistically significant further reduction in tensile or yield strength occurs between 12 h and 24 h. After 24 h of thermal exposure, the tensile strength and yield strength of the base alloy decreased by approximately 53.36% and 66.99%, respectively. In contrast, the tensile strength and yield strength of the 4.5 wt.% Al_3_Zr composite decreased by approximately 47.31% and 64.99%, respectively, both of which were smaller than those of the base alloy. Figure 13c illustrates the variation in microhardness during thermal exposure. The microhardness of both materials decreased significantly and continuously with prolonged exposure time, each exposure duration forming a distinct letter group (0 h > 12 h > 24 h, *p* < 0.05). Throughout the thermal exposure period, the composite consistently maintained a higher hardness than the base alloy; the hardness gap widened from approximately 7.56 HV in the unexposed state to approximately 18.97 HV after 24 h.

Based on the comprehensive analysis of microstructural and mechanical property results, it is evident that under prolonged thermal exposure at 350 °C, the θ′ phase underwent significant coarsening and gradually transformed toward the equilibrium θ-Al_2_Cu phase, leading to a progressive weakening of the precipitation strengthening effect and consequently resulting in decreases in tensile strength, yield strength, and microhardness. However, the Al_3_Zr particles maintained stable dimensions and morphologies throughout the thermal exposure process, enabling them to exert dispersion strengthening and dislocation pinning effects at elevated temperatures, thereby effectively retarding the deterioration of material properties. Therefore, the superior elevated-temperature stability of the composite is governed primarily by the dimensional stability of the Al_3_Zr particles, which retain their dispersion-strengthening and dislocation-pinning effects throughout exposure. The smaller degree of θ′ coarsening observed in the composite is a secondary contributing factor whose mechanism is not fully resolved by the present measurements; together, these effects render the composite more resistant to thermal-exposure softening than the base alloy.

## 4. Conclusions

In this study, *x*Al_3_Zr/Al-5Cu-0.6Mn-0.15Ti composites were synthesized through an in situ reaction technique. This study investigated how varying Al_3_Zr additions affect the microstructure changes and tensile properties under both room-temperature and elevated-temperature conditions, using specimens in the as-cast and T6-treated states. The key findings are outlined below.

1. The *x*Al_3_Zr/Al-5Cu-0.6Mn-0.15Ti composites were successfully fabricated via an in situ reaction between K_2_ZrF_6_ and molten aluminum. The microstructures were primarily composed of α-Al, Al_2_Cu, Al_12_CuMn_2_, and Al_3_Zr phases, among which the in situ formed Al_3_Zr exhibited a D0_23_-type tetragonal structure. Microstructural analysis of the as-cast composites revealed that the α-Al grains were progressively refined with increasing Al_3_Zr content. Upon T6 heat treatment, numerous finely dispersed metastable θ′-Al_2_Cu precipitates were formed within the matrix, and the Al_3_Zr particles underwent edge blunting and spheroidization. The grain size of all alloys increased; however, the magnitude of grain growth in the composites was considerably lower than that in the base alloy.
2. The incorporation of Al_3_Zr particles enhanced the tensile properties of the composites. Under room-temperature conditions, both the as-cast and T6-treated composites achieved their maximum tensile strengths at 4.5 wt.% Al_3_Zr. During elevated-temperature tensile testing at 350 °C, the as-cast composites attained their highest tensile strength and yield strength at 6 wt.% Al_3_Zr, whereas the T6-treated composites achieved their maximum tensile strength and yield strength at 4.5 wt.% Al_3_Zr. Under as-cast conditions, the strength enhancement was primarily attributed to grain-refinement strengthening of α-Al together with the load-transfer and dispersion strengthening provided by the Al_3_Zr particles.
3. Under prolonged thermal exposure at 350 °C, the θ′-Al_2_Cu precipitates in both the base alloy and the Al_3_Zr-containing composite underwent coarsening, weakening the precipitation-strengthening effect and producing continuous decreases in tensile strength, yield strength, and microhardness. The 4.5 wt.% Al_3_Zr composite showed a smaller degree of θ′ coarsening and smaller reductions in strength and hardness than the base alloy. Because the Al_3_Zr particles remained dimensionally stable throughout exposure, this improved thermal-exposure resistance is attributed mainly to the persistence of their dispersion-strengthening contribution; the reduced θ′ coarsening is an additional contributing factor, the precise mechanism of which warrants further interface-resolved investigation.

## Figures and Tables

**Table 1:** Measured chemical compositions of the *x*Al_3_Zr/Al-Cu-Mn-Ti composites (wt.%).

Alloy	Al	Cu	Mn	Ti	Zr	Fe	Si
Base alloy	Bal.	4.87	0.57	0.12	0.00	0.16	0.00
1.5 wt.% Al_3_Zr composite	Bal.	4.7	0.53	0.16	0.68	0.07	0.02
3 wt.% Al_3_Zr composite	Bal.	4.68	0.57	0.14	1.43	0.17	0.01
4.5 wt.% Al_3_Zr composite	Bal.	4.81	0.62	0.12	2.27	0.08	0.03
6 wt.% Al_3_Zr composite	Bal.	4.83	0.64	0.17	3.05	0.13	0.03

**Table 2:** Quantitative statistical results of the microstructure of as-cast Al_3_Zr/Al-5Cu-0.6Mn-0.15Ti composite material.

Alloy	α-Al		θ-Al*2*Cu		Al*3*Zr	
	Particle Size (μm)	Area Fraction (%)	Particle Size (μm)	Area Fraction (%)	Particle Size (μm)	Area Fraction (%)
0 wt.% Al_3_Zr	72.95 ± 23.97	94.83	32.79 ± 11.14	5.24	-	-
1.5 wt.% Al_3_Zr	50.99 ± 17.51	93.43	28.41 ± 8.91	5.10	1.87 ± 0.37	0.84
3 wt.%Al_3_Zr	43.57 ± 11.98	90.16	23.54 ± 6.14	5.06	1.72 ± 0.54	4.38
4.5 wt.% Al_3_Zr	37.09 ± 11.02	88.72	21.50 ± 6.39	4.75	1.38 ± 0.43	5.54
6 wt.% Al_3_Zr	38.04 ± 10.21	88.34	21.76 ± 4.79	4.87	2.21 ± 0.75	5.81

**Table 3:** Quantitative statistical results of the microstructure of heat-treated Al_3_Zr/Al-5Cu-0.6Mn-0.15Ti composite materials.

Alloy	α-Al		θ-Al_2_Cu		Al_3_Zr	
	Particle Size (μm)	Area Fraction (%)	Particle Size (μm)	Area Fraction (%)	Particle Size (μm)	Area Fraction (%)
0 wt.% Al_3_Zr	74.50 ± 27.46	94.24	19.34 ± 9.18	1.61	-	-
1.5 wt.% Al_3_Zr	58.00 ± 23.17	93.62	15.67 ± 6.06	1.75	2.58 ± 0.42	0.64
3 wt.% Al_3_Zr	53.26 ± 19.28	91.18	13.39 ± 5.27	1.54	2.45 ± 0.38	3.86
4.5 wt.% Al_3_Zr	46.66 ± 15.03	90.69	13.00 ± 4.83	1.52	2.09 ± 0.35	4.57
6 wt.% Al_3_Zr	44.39 ± 13.91	90.24	15.35 ± 6.94	1.48	2.95 ± 0.52	5.14

**Table 4:** Comparison of ultimate tensile strength with other heat resistant aluminum alloys.

Material	Temperature/°C	UTS/MPa	Year	References
4.5 wt.% Al_3_Zr/Al-5Cu-0.6Mn-0.15Ti	350	123.38	2025	Our work
Al-5.47Si-0.62Cu-1.21Mn-1.71Ni-0.1Mg-0.42Ti	350	98.9	2025	[43]
Al-12.1Si-0.89Cu-0.49Mn-3.95Ni-0.68Mg-0.17Ti	350	99.3	2025	[43]
Al-11.69Si-0.19Cu-0.3Mn-1.61Ni-1.01Mg-0.79Fe	350	108.71	2025	[43]
Al-12.5Si-1Cu-0.3Zr-0.4V	350	79.4	2021	[44]
(2 Al_3_Zr + 15.2 Al_3_Ni)/Al-1Mg-0.8Mn-0.8V	350	82	2019	[45]
Al-12Si-4Cu-2Ni-1Mg-AlNp	350	106	2019	[46]
Al-12Si-4Cu-2Ni-1Mg	350	85	2019	[46]
Al-12.95Si-3.57Cu-0.72Mg-0.91Ni-0.53Fe-0.4Er	350	117	2019	[47]
Al-12Si-3.5Cu-2Mn-1Cr	350	106	2018	[48]
Al-11.79Si-3.33Cu-0.172Fe-2.05Mn-1Cr	350	106	2018	[48]
Al-13Si-4Cu-2Ni-1Mg-0.25Mn	350	92	2018	[48]
Al-12.01Si-3.53Cu-0.189Fe-2.12Mn	350	83	2018	[48]
Al-12.21Si-3.42Cu-0.192Fe-2.02Mn-0.5Cr	350	95	2018	[48]
Al-11.98Si-3.38Cu-0.188Fe-2.01Mn-1.5Cr	350	91	2018	[48]
Al-12Si-0.9Cu-0.8Mg-*x*Ni	350	116	2017	[49]
Al-12.5Si-0.84Mg-5Cu-2Ni-0.5Fe-(0.24~0.28) Cr	350	92	2015	[50]

## Data Availability

The original contributions presented in this study are included in the article. Further inquiries can be directed to the corresponding author.
